# Perceptual sensitivity is modulated by what others can see

**DOI:** 10.3758/s13414-019-01724-5

**Published:** 2019-05-06

**Authors:** Tricia Seow, Stephen M. Fleming

**Affiliations:** 1 Wellcome Trust Centre for Neuroimaging, University College London, 12 Queen Square, London, WC1N 3BG UK; 2 Department of Psychology, Trinity College Dublin, Dublin, Ireland; 3 Max Planck UCL Centre for Computational Psychiatry and Aging Research, University College London, 10-12 Russell Square, London, WC1B 5EH UK

**Keywords:** Attention, Bayesian modeling, Signal detection theory

## Abstract

Previous work has established that social cues such as the direction of others’ gaze or their perspective on a scene may influence one’s own perceptual judgments. However, up until now it has remained unclear whether such influences are exerted at a perceptual or decisional locus, as most previous studies have used response times as their primary dependent measure. Here, we asked whether perceptual sensitivity is also dependent on social cognition. To test this hypothesis, we asked participants to evaluate whether low-contrast Gabor patterns embedded in noise were visible from either their own or an avatar’s perspective. Across three experiments, we found that observers’ detection performance was increased if an avatar also shared perception of the stimulus location. By leveraging signal detection modelling, we show that this effect is driven by a change in perceptual sensitivity (*d′*), independent of decisional or response interference. Furthermore, by “blindfolding” the avatar, we show that the boosting effect of shared perception on detection sensitivity is only obtained when the participant believes the avatar can also see the stimulus, ruling out an influence of low-level directional cues. We interpret these results within a framework in which the avatar’s perspective boosts top-down spatial attention by prioritising particular spatial locations at which perception is shared. In summary, we reveal that perceptual sensitivity is modulated by the perspective of others.

**Electronic supplementary material:**

The online version of this article (10.3758/s13414-019-01724-5) contains supplementary material, which is available to authorized users.

Whether a stimulus is consciously perceived is determined by the strength of sensory processing and top-down factors such as expectations and attention (Dehaene & Changeux, 2011; Summerfield & Egner, 2009). Signal detection theory (SDT) provides a framework for decomposing perceptual performance into two statistics: *d'*, the sensitivity of the system to signal occurrence in signal-to-noise ratio (SNR) units, and an overall bias to report signal presence, modelled as the criterion, *c* (Green & Swets, 1966; Macmillan & Creelman, 2005). It is well established that simple attentional cues predicting behaviourally relevant spatial locations lead to increased sensitivity (Carrasco, Ling, & Read, 2004; Müller & Findlay, 1987; Wyart, Nobre, & Summerfield, 2012). In parallel, previous work has shown that social cues such as the direction of others’ gaze or their visual perspective on a scene can influence one’s own judgments and decisions (Cole, Smith, & Atkinson, 2015; Furlanetto, Becchio, Samson, & Apperly, 2016; Qureshi, Apperly, & Samson, 2010; Samson, Apperly, Braithwaite, Andrews, & Bodley Scott, 2010; Wiese, Wykowska, Zwickel, & Müller, 2012). For instance, when the number of stimuli visible to the subject and an avatar are in conflict, response times to verify the number are slowed (Samson et al., 2010). However, it remains unknown whether sharing perception of a scene with others affects perceptual sensitivity, as most previous studies of visual perspective-taking have used response times (RTs) as their primary dependent measure.

Here we asked whether perceptual sensitivity is also dependent on the perspective of others. In other words, when looking at a sunset with a friend, is the sunset perceived differently than if you were alone? Or, more prosaically, are you more likely to detect a target if your co-observers can see it too? To answer this question, we manipulated whether a virtual avatar did or did not share the participant’s view of low-contrast Gabor patterns embedded in noise (see Fig. 1a). On each trial, the Gabor was either congruent (visible to both the avatar and the participant), incongruent (not visible to the avatar, but visible to the participant), or absent. Before the trial began, participants were cued to report the visibility of the Gabor from either their own (“self”) or the avatar’s (“other”) perspective. Although criterion shifts cannot be unequivocally tied to a perceptual or decisional locus (Morgan, Hole, & Glennerster, 1990; Witt, Taylor, Sugovic, & Wixted, 2015), changes in sensitivity are typically modelled as changes in signal-to-noise ratio, indicating selective effects on visual perception independent of response biases (Green & Swets, 1966; Macmillan & Creelman, 2005; Witt et al., 2015).

**Fig. 1:**
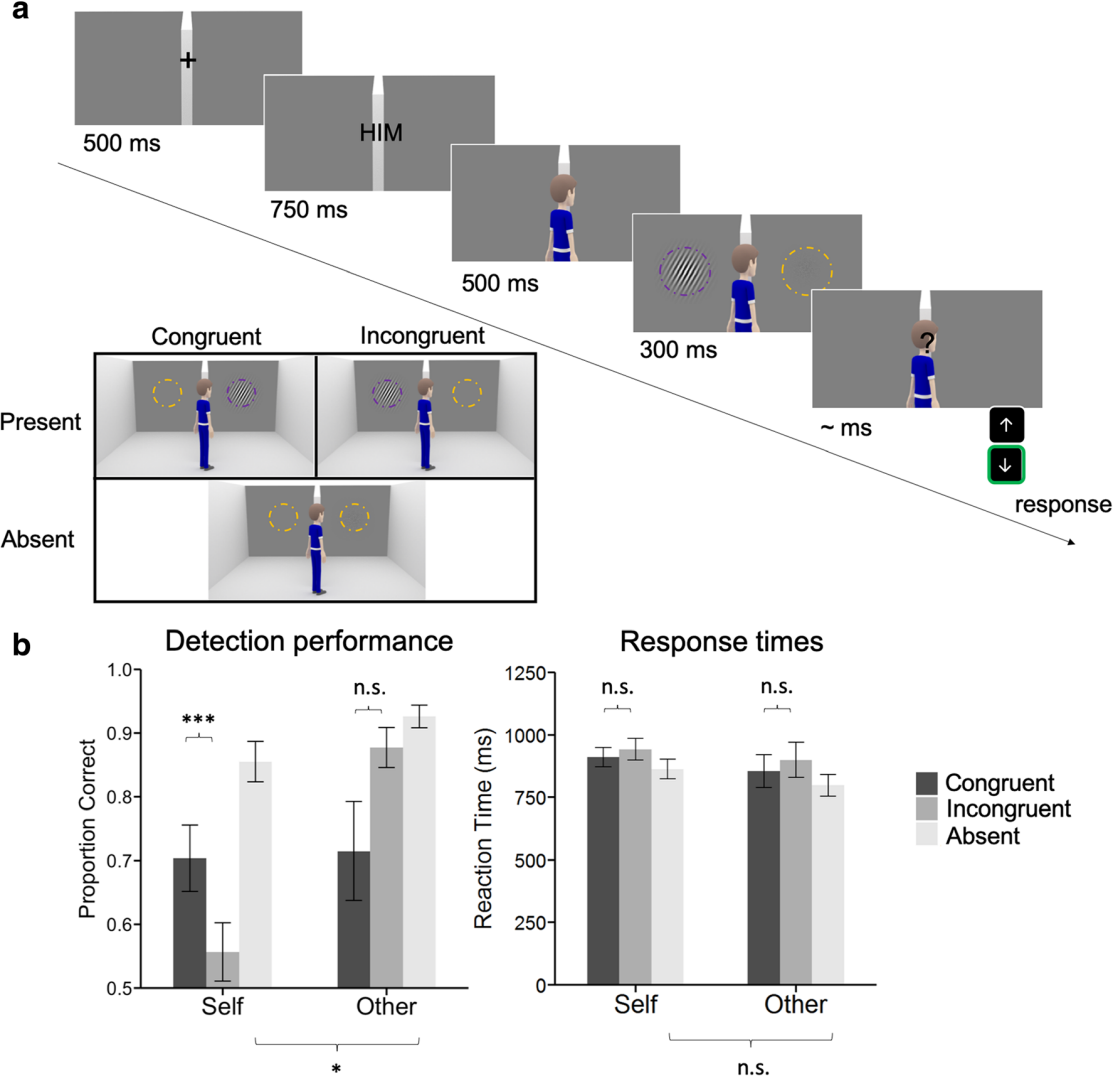
**a** Experimental design and stimuli. On each trial, subjects were cued as to which perspective they should adopt for that particular trial. A male avatar oriented to one or the other half of the room then appeared, followed by briefly presented low-contrast Gabor(s)/noise patch(es). Participants were asked to judge whether a Gabor could be seen from the relevant perspective (“Him” or “You”) with a button press. Gabors are indicated with dashed purple circles, and noise patches with dashed orange circles, neither of which were present in the experiment. In Experiment 1, either two noise patches or one noise patch and one Gabor were presented. Examples of trials in the different experimental conditions are shown in the inset: Congruent = the Gabor was visible to both the avatar and observer; Incongruent = the Gabor was visible to the observer, but not the avatar; Absent = only noise patches were presented, and thus congruency was not defined. In all panels, the background is cropped and stimuli enlarged for presentation purposes. **b** Visual detection performance in Experiment 1. Each panel indicates the accuracy and response times of visual detection judgments sorted by condition. Symbols indicate the significance of paired *t* tests (*p < .05. **p < .01. ***p < .001). Error bars indicate *SEM* (*N* = 10)

We used the same paradigm, with slight modifications, across a series of three experiments. In all experiments, the contrast of the target Gabor was titrated for each individual participant to achieve ~70% detection accuracy in an initial thresholding session (see Experiment 1, Method, for details), and subsequently kept constant throughout the experiment. To facilitate SDT analysis, in all experiments we excluded subjects who performed <60% or >90% correct in the critical “self” condition from further analysis (see Experiment 2, Method). Our key findings nevertheless hold when all subjects are included (see Supplementary Materials).

The extent to which the avatar’s perspective interferes with participants’ perceptual sensitivity was estimated by analysing performance on the critical “self” trials. Our central hypotheses focus on differences between congruent and incongruent trials within the “self” condition (when the avatar’s perspective is irrelevant for the observer’s decision) to investigate whether visual detection is affected by the perspective of others. We also conducted exploratory analyses to understand the pattern of results obtained in the “other” condition. We report these in a separate results section before the General Discussion.

In Experiment 1, we explore the effect of sharing perception with the avatar on visual detection. In Experiment 2, we modified the design to allow SDT analysis to separately quantify the influence of the avatar on changes in perceptual sensitivity and the criterion for detection. Lastly, in Experiment 3, we demonstrate that the effect of the avatar on perceptual sensitivity is underpinned by beliefs about shared perception rather than reflecting the influence of low-level directional cues.

## Experiment 1

### Method

#### Power simulations

Previous work utilising classic dot perspective tasks investigating the effect of self and other perspectives on judgements of explicitly visible stimuli reported a very large main effect of congruency on response times, *F*(1, 15) = 45.51, *MSE* = 2,127.31, *p* < .001, η^2^_p_ = 0.752 (Samson et al., 2010). This effect size suggests that only ~*N* = 4 is required to achieve 95% power. However, as our study utilized novel near-threshold stimuli and perceptual sensitivity measures with unknown effect sizes, we decided to collect data from a larger pool of ~*N* = 20.

#### Participants

Participants across all three experiments had normal or corrected-to-normal vision, recruited from the University College London (UCL) psychology subject pool and provided informed consent. The study was approved by the UCL Research Ethics Committee.

Nineteen participants (16 females, mean age = 27 years, *SD* = 9.13, range: 19–53 years) took part in Experiment 1. We excluded any participant for whom mean accuracy on “self” trials was >90% or <60% correct to avoid ceiling/floor effects on detection performance. Nine participants were excluded, leaving 10 for analysis (seven females; mean age = 27.6 years, *SD* = 7.91, range: 20–41 years).

#### Stimuli and procedure

The background and avatar were generated with the three-dimensional animation software Blender (Version 2.76; https://www.blender.org/) and stimulus presentation was controlled by Cogent 2000 and Cogent Graphics (http://www.vislab.ucl.ac.uk/cogent.php) in MATLAB (The MathWorks, Natick, MA).

Experimental stimuli were either noise patches or Gabor patches (a circular patch of sinusoidal light and dark bars). Noise patches consisted of randomly generated white noise at 10% contrast and modulated by a Gaussian envelope. Gabor patches consisted of sinusoidal gratings (spatial frequency of five cycles per degree, orientation 30 degrees), superimposed with 10% white noise and modulated by a Gaussian envelope. Both Gabors and noise patches subtended ~3 degrees of visual angle, and were presented at an eccentricity of ~4.5 degrees. Gabor contrast was determined as described below, and all luminance increments were gamma corrected. Subjects were asked to press either the up key on a standard keyboard if the Gabor was visible from the relevant perspective, or the down key if it was absent.

Participants were seated approximately 60 cm away from a Dell Latitude E5550 series laptop running at a resolution of 1,024 × 768 pixels and situated in a darkened room. Prior to the start of the main experimental session, Gabor contrast (the luminance difference between light and dark bars) was calibrated for each individual participant using the QUEST procedure (Watson & Pelli, 1983) to estimate a Gabor contrast that yielded 70% correct performance in a two-interval forced-choice task. Three independent threshold estimates were acquired, with 40 randomly ordered trials contributing to each. The mean value of these threshold estimates was used to set the contrast of the Gabor in the main experiment, which then remained constant.

In the main experiment, the background consisted of a slightly elevated frontal view of a 3-D room with a grey back wall split by a white divider. A male human avatar was positioned in the centre of the screen looking forwards into the room, either to the left or right side of the divider. Each trial began with a fixation cross presented for 500 ms (see Fig. 1). The word “YOU” or “HIM” was then shown for 750 ms, indicating which perspective (“self” or “other”, respectively) participants should take for that particular trial. The avatar then appeared for 500 ms followed by stimuli superimposed on the grey wall of the room for 300 ms. Participants then indicated with a button press whether a Gabor was present (“Yes” or “No” response) from the perspective they were instructed to adopt in that particular trial.

In Experiment 1, stimuli composed of either two noise patches (Gabor-absent trials) or one noise patch and one Gabor patch (Gabor-present trials). Participants performed four blocks of 100 trials in a factorial design crossing perspective = self/other, avatar direction = left/right, stimulus position = left/right and Gabor = present/absent. As two noise patches were presented on Gabor-absent trials, we were unable to define a stimulus position for “absent” trials. Trial order was randomised.

Congruency refers to the relationship between Gabor location and avatar direction. Trials were labelled as congruent if Gabor location was the same as avatar direction (as both the participant and avatar were able to see the Gabor; a shared perspective) and incongruent if the Gabor location was not the same as avatar direction (i.e., the Gabor was only visible to the participant). Thus, there were three conditions in Experiment 1: congruent (where the avatar could see the Gabor), incongruent (where the avatar could not see the Gabor) and absent (only noise patches presented, and congruency is undefined). The three conditions of Experiment 1 are illustrated in Fig. 1a.

The correct response as to whether a Gabor was visible for each trial thus depended on the cued perspective, Gabor presence and congruency for that trial. For instance, if the participant sees the word “YOU” (“self” condition; they should take their own perspective), the avatar is irrelevant for the response in this condition as the participant is able to see the Gabor regardless of where it appears relative to the dividing wall. Thus in a Gabor-present trial, they should indicate that a Gabor was present (“Yes” response), while in a Gabor-absent trial, they should indicate that a Gabor was absent (“No” response).

However, if the participant sees the word “HIM” (“other” condition; they should take the avatar’s perspective), the avatar is important for determining the response in this condition as the avatar is only able to “see” the Gabor on the same side of the wall to where he is looking. Thus, in Gabor-present-congruent trials, the participant should indicate that a Gabor is present from the avatar’s perspective (“Yes” response). In contrast, if a Gabor appears on the side opposite to where the avatar is looking (incongruent trials) or is absent, they should indicate that a Gabor is absent from the avatar’s perspective (“No” response).

#### Data preprocessing

Response times (RTs) were measured from stimulus onset. Individual trials were excluded from analysis if log response times were in excess of three standard deviations of the participant’s mean log(RT). A mean of 3.9 trials per subject were excluded.

### Results

In Experiment 1, we analysed data from *N* = 10 observers who reported whether near-threshold Gabor patches were visible from either their own or an avatar’s perspective (see Fig. 1). Critically, on Gabor-present “self” trials, participants were more likely to successfully detect the Gabor when it was also visible to the avatar (leftmost bars of Fig. 1b), *t*(9) = 4.78, *p* < .001. To further quantify the effect of congruency and perspective type on behavioural performance, we performed a 2 × 2 repeated-measure analysis of variance (ANOVA) on responses to Gabor-present trials with congruency (congruent/incongruent) and perspective type (self/other) as within-subjects factors. We found a significant Congruency × Perspective interaction, *F*(1, 9) = 14.85, *p* < .001, for accuracy, due to a shared perspective boosting detection on “self” but not “other” trials. Effects on detection accuracy were obtained in the absence of changes in response time, suggesting a lack of decisional or response interference (see Fig. 1b, right panel): There were no response-time main effects of congruency, *F*(1, 9) = 2.77, *p* = .13, perspective; *F*(1, 9) = 3.08, *p* = .11; or a Congruency × Perspective interaction, *F*(1, 9) = 0.163, *p* = .70, for Gabor-present trials. Gabor-absent trials also showed no effect of perspective on response time, *t*(9) = 1.24, *p* = .25. Taken together, these results indicate that visual detection performance on critical Gabor-present “self” trials is boosted by shared perception.

### Discussion

We reasoned that the effect of the avatar on visual detection observed in Experiment 1 may be due either to a change in response bias (an increased tendency to report Gabor presence at locations viewed by the avatar, irrespective of the stimulus condition), an increase in perceptual sensitivity, or both. It was not possible to discriminate between these alternatives in Experiment 1, because congruency was undefined for Gabor-absent trials in which a pair of noise patches was always presented. Additionally, while we aimed to collect data from *N* = 20 subjects, the QUEST calibration procedure was not as precise as we had hoped. As such, several participants deviated from the target accuracy in the main experiment resulting in a relatively small sample size after exclusion criteria were applied.

To evaluate the possibilities outlined above and to replicate our initial findings in a larger sample, we carried out a second experiment (*N* = 18, after exclusions) in which a single noise patch or Gabor was presented on either side of the central dividing wall. This design alteration allowed congruency to be defined on both Gabor-present and Gabor-absent trials (i.e., a noise patch that was either visible or invisible to the avatar), permitting calculation of the effect of the avatar’s perspective on both hits (“Yes” responses when the Gabor was present) and false alarms (“Yes” responses when the Gabor was absent).

## Experiment 2

### Method

#### Participants

Twenty-nine participants (20 females, mean age = 24 years, *SD* = 6.53, range: 18–52 years) took part in Experiment 2. Eleven participants were excluded with the same exclusion criteria as Experiment 1, leaving 18 for analysis (15 females; mean age = 22.6 years, *SD* = 4.38, range: 18–37 years).

#### Stimuli and procedure

We used the same stimuli and procedure as in Experiment 1, except that only one stimulus (either a noise patch or Gabor patch) was presented on each trial. Participants again performed four blocks of 100 trials in a fully factorial design crossing perspective = self/other, avatar direction = left/right, stimulus position = left/right and Gabor = present/absent.

**Fig. 2:**
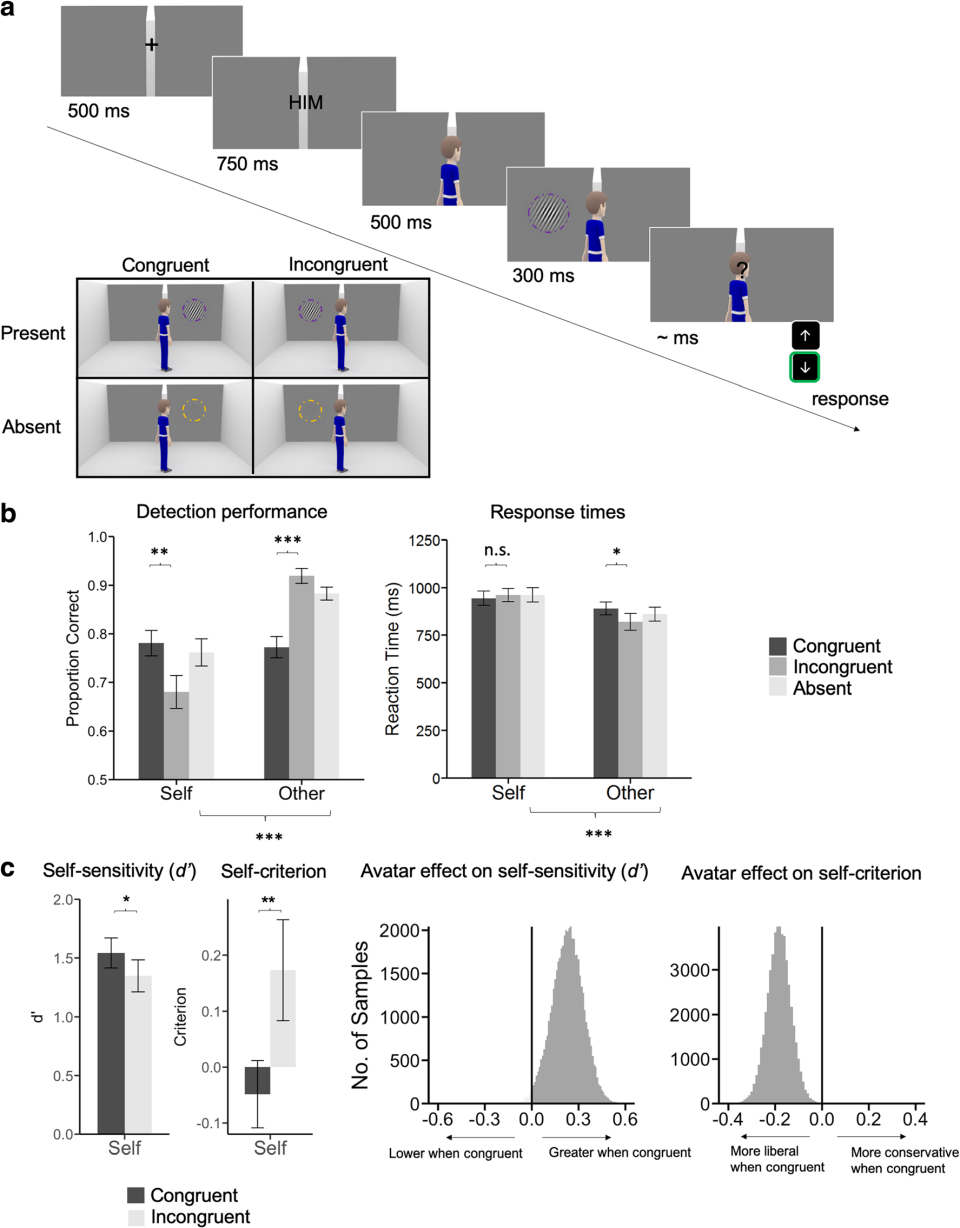
**a** Design and stimuli of Experiment 2 were similar to Experiment 1, except that only one stimulus was presented on each trial (either a noise patch or a Gabor, to the left or right of the divider). Examples of trials in the different experimental conditions are shown in the inset; congruency is now defined for both Gabor-absent and Gabor-present trials, as only one noise patch was presented on each Gabor-absent trial. In all panels, the background is cropped and stimuli enlarged for presentation purposes. **b** Mean accuracies and response times in Experiment 2, plotted using the same conventions as in Fig. 1b. **c** Mean *d′* and criterion for the “self” trials as well as posterior densities of the difference between congruent and incongruent conditions for perceptual sensitivity (*d′*) and bias (criterion). The change in colour indicates where the distribution crosses the zero point. Symbols indicate the significance of paired *t* tests (*p < .05. **p < .01. *** *p* < .001). Error bars indicate *SEM* (*N* = 18)

In contrast to Experiment 1, stimulus position was now relevant on both noise trials (when a single noise patch was presented) and signal trials (when a Gabor patch was presented), which was crucial to allow a factorial analysis of sensitivity. Thus, congruency referred to the relationship between stimulus location (Gabor or noise patch) and the direction the avatar is looking. There were four conditions for Experiment 2: Gabor-present congruent (avatar could see the Gabor), Gabor-present incongruent (avatar could not see the Gabor), Gabor-absent congruent (avatar could see the noise patch) and Gabor-absent incongruent (avatar could not see the noise patch; see Fig. 2a).

#### Data preprocessing

Data of Experiment 2 were preprocessed in the same way as for Experiment 1. A mean of 3.4 trials per subject were excluded.

#### Signal-detection analysis

Responses on “self” trials were sorted into hits, false alarms, misses, and correct rejections (this analysis was not applied to Experiment 1, as it was not possible to determine congruency on Gabor-absent trials). For each subject, detection sensitivity (*d'*) and criterion (*c*) were calculated separately for congruent and incongruent trials as follows:

$$ {d}^{\prime }=z(H)-z(FA)c=-0.5\times \left[z(H)+z(FA)\right] $$

where *z* indicates the inverse of the cumulative normal distribution, *H* is the hit rate, and *FA* is the false-alarm rate. We additionally fitted a hierarchical Bayesian signal detection model (Lee, 2008) to obtain posterior distributions of group-level sensitivity and bias parameters. We used Markov chain Monte Carlo (MCMC) as implemented in JAGS in R (Plummer, 2003) to draw samples from the posterior distributions. JAGS was called with 2,000 adaptation steps, 5,000 burn-in samples and 50,000 effective samples. Three chains for each parameter were run, and convergence of all chains was assessed both visually and using Gelman and Rubin’s potential scale-reduction statistic $\widehat{R}$ for all parameters (Gelman & Rubin, 1992). Large values of $\widehat{R}$ indicate convergence problems and values ~1 suggest convergence. Average $\widehat{R}$ was 1.00, and all values were <1.1, indicating good convergence. We fitted the model described in Lee (2008) twice, once for each of the congruent and incongruent conditions.

The posterior distributions of each parameter returned by JAGS can be straightforwardly employed for Bayesian inference (Kruschke, 2014). For example, to assess the difference between two conditions, congruent and incongruent, we can directly calculate the probability that the difference between the two parameters is larger than zero, ${P}_{\theta}\left({d}_{congruent}^{\prime }-{d}_{incongruent}^{\prime }>0\right)$, where a high probability indicates strong evidence in favour of a difference. We denote these probabilities as *P*_*θ*_ to distinguish them from classical *p* values.

### Results

Participants were again more likely to successfully detect the Gabor when it was also visible to the avatar on “self” trials (see Fig. 2b), *t*(17) = 3.63, *p* = .002, with no change in response time, *t*(17) = −0.90, *p* = .38, thereby replicating our key finding from Experiment 1. Classical paired *t* tests revealed that congruent (vs. incongruent) avatar perspectives boosted *d'*, *t*(17) = 2.35, *p* = .03, and led to a more liberal criterion, *t*(17) = −3.53, *p* = .003. Examination of hit and false-alarm rates showed that this effect was due to a selective increase in hit rate on congruent trials, *t*(17) = 3.63, *p* = .002, in the absence of any change in false-alarm rate, *t*(17) = 0.98, *p* = .34 (see Fig. S1 in the Supplementary Materials). As in Experiment 1, no significant effects of condition were observed on response times between “self” congruent and incongruent trials, *t*(17) = −0.57, *p* = .57 (see Fig. 2b). However, in this case an ANOVA revealed significant main effects of congruency, *F*(1, 17) = 7.54, *p* = .01; perspective, *F*(1, 17) = 38.13, *p* < .001; and a Congruency × Perspective interaction, *F*(1, 17) = 11.88, *p* = .003, due to faster responses on “other” incongruent trials.

We additionally fitted a hierarchical Bayesian signal-detection model to all subjects’ data to obtain group-level posterior distributions of sensitivity (*d'*) and criterion (*c*) separately for congruent and incongruent “self” trials (see Experiment 2, Method). We compared group-level posteriors using a Bayesian approach that returns a probability in support of the alternative hypothesis (Kruschke, 2014), which we denote as *P*_*θ*_ to distinguish it from a classical *p* value. This analysis revealed effects of shared perception on both perceptual sensitivity and detection criterion (see Fig. 2C and Supplementary Materials’ Fig. S7; both *P*_*θ*_s > .99). Taken together, the results of Experiment 2 indicate a selective increase in perceptual sensitivity is obtained when the avatar’s perspective is aligned with that of the participant (results on “other” trials are presented in a separate section below).

### Discussion

**Fig. 3:**
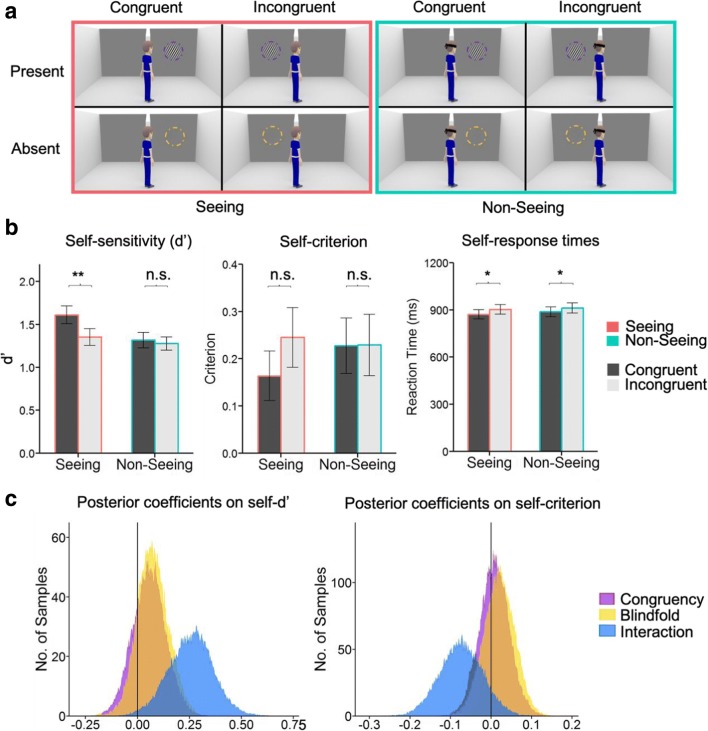
**a** Schematic of trials in the different conditions of Experiment 3. In the nonseeing conditions, the avatar wore a blindfold. This factor was crossed in a fully factorial design with perspective, congruency, and Gabor presence. **b** Mean *d′*, criterion, and response times for “self” trials in Experiment 3. Symbols indicate the significance of paired *t* tests (*p < .05. **p < .01. ***p < .001). Error bars indicate *SEM* (*N* = 37). **c** Posterior densities of estimated regression coefficients for the effects of congruency, blindfold, and their interaction on perceptual sensitivity (*d′*) and bias (criterion). Vertical line indicates the zero point. (Colour figure online)

We next considered whether the effects of the avatar on perceptual sensitivity on “self” trials could be due to low-level directional cues rather than beliefs about shared perception (Heyes, 2014). For instance, simply by being oriented to one or other side of the room, the avatar may act as a directional cue towards congruent Gabors (Driver et al., 2010; Guzzon, Brignani, Miniussi, & Marzi, 2010; Posner, 1980). Such directional cueing may occur in the absence of any assessment of whether the avatar can or cannot see the stimulus. Following Heyes (2014), we reasoned that if the effect of the avatar on visual detection was only due to a directional cue, it would remain intact if the avatar’s directional features were maintained, but he was unable to see due to being blindfolded (see Fig. 3a). If, on the other hand, the effects observed in Experiments 1 and 2 were mediated by shared perception of the stimulus, they should be abolished when the avatar is unable to see. In a new cohort of participants (*N =* 37), we extended the design of Experiment 2 such that on half the trials the avatar was blindfolded, resulting in a 2 (self, other) × 2 (blindfold, no blindfold) × 2 (congruent, incongruent) experimental design (see Fig. 3a).

## Experiment 3

### Method

#### Power simulations

To ensure that Experiment 3 was well powered despite the added complexity of the design, we carried out SDT simulations of our experimental hypothesis that the effect of congruency would only be obtained on seeing trials. First, we drew values of baseline *d'* and criterion from the group-level posterior of the baseline parameters from Experiment 2. For each run of the simulation, this draw was assigned to the conditions congruent/nonseeing, incongruent/nonseeing, and incongruent/seeing. To create the increased *d'* and more liberal criterion expected in the congruent/seeing condition, we drew a sample from the posterior of the difference between congruent and incongruent conditions in Experiment 2, and added this to each baseline parameter.

SDT parameters for each condition were then used to generate hit and false-alarm counts from a binomial observation model with 96 trials per condition, and the observed *d'* and criteria were calculated. This procedure was repeated for each simulated subject, and a repeated-measures ANOVA was applied to obtain a p value for the Seeing × Congruency interaction effect. We repeated the simulations 1,000 times for sample sizes ranging from *N* = 10 to *N* = 50, and stored the power to detect a significant interaction effect at *p* < .05. For *d'*, 80% power was obtained at a sample size of *N* = 45; for criterion, 80% power was obtained at a sample size of *N* = 20. We collected data from *N* = 46 subjects in Experiment 3.

#### Participants

Forty-six participants (28 females, mean age = 26 years, *SD* = 8.82, range: 18–65 years) took part in Experiment 3. Nine participants were excluded with the same exclusion criteria as in Experiments 1 and 2, leaving 37 for analysis (27 females; mean age = 24.35 years, *SD* = 5.97, range: 18–47 years).

#### Stimuli and procedure

The same stimuli and procedure were used as in Experiment 2, with an additional condition: In half of the trials, the avatar was blindfolded. Participants were instructed that the blindfolded avatar would not be able to see the stimuli. Experiment 3 thus comprised seven blocks of 96 trials in a fully factorial design crossing perspective = self/other, avatar direction = left/right, stimulus position = left/right, Gabor = present/absent, and blindfold = seeing/nonseeing (Fig. 3a). The correct response on “other” trials was determined by whether or not the avatar was able to see the Gabor. Thus, in all nonseeing “other” trials, the correct answer was a “No” response.

#### Data preprocessing

Data of Experiment 3 were preprocessed in the same way as for Experiments 1 and 2. A mean of 5.3 trials per subject were excluded.

#### Signal-detection analysis

Similar to Experiment 2, responses on “self” trials were sorted into hits, false alarms, misses and correct rejections. For each subject, detection sensitivity (*d'*) and criterion (*c*) were calculated separately for each cell of the 2 (seeing, nonseeing) × 2 (congruent, incongruent) factorial design. We nested the hierarchical SDT model inside a linear regression model that encoded the two factors of our experimental design (Blindfold × Congruence), plus their interaction. Thus each subject’s *d'* parameter was specified as:

$$ {d}^{\prime }={d}_{base}^{\prime }+{\beta}_c\ast {I}_c+{\beta}_b\ast {I}_b+{\beta}_i\ast {I}_c\ast {I}_b $$

where *I*_*c*_ and *I*_*b*_ are indicator variables that are equal to 1 when the condition is congruent/seeing and 0 otherwise, and *β*_*c*_, *β*_*b*_ and *β*_*i*_ are regression coefficients encoding the effects of congruency, blindfold and their interaction, respectively. Uninformative (high variance) priors on these influences on *d'* were specified as follows (after JAGS convention, variances are written as precisions, or the reciprocal of variance):

$$ {d}_{base}^{\prime}\sim N\left(0,0.001\right) $$

$$ {\beta}_c\sim N\left(0,0.001\right) $$

$$ {\beta}_b\sim N\left(0,0.001\right) $$

$$ {\beta}_i\sim N\left(0,0.001\right) $$

Analogous regression parameters and priors were applied to estimate the influence of each experimental factor on the criterion, *c*.

### Results

#### Boosts in perceptual sensitivity are dependent on shared perception

In Experiment 3, due to the added complexity of the design, we increased the trial number (672 trials per subject) and doubled our sample size to ensure sufficient power (see Experiment 3, Method, for details). Focusing on the critical “self” trials in which the avatar’s perspective was irrelevant for the detection judgment, we quantified the influence of these factors and their interaction on SDT parameters *d'* and *c*.

Both congruency (*P*_*θ*_ = .77) and blindfold (*P*_*θ*_ = .84) engendered positive effects on *d'* such that greatest perceptual sensitivity was observed in the seeing, congruent condition (leftmost bar in left panel of Fig. 3b). Critically, these main effects were qualified by a significant Congruency × Blindfold interaction (see Fig. 3b–c; *P*_*θ*_ = .99) due to congruent (vs. incongruent) avatar perspectives boosting *d'* in the seeing condition, *t*(36) = 2.93, *p* = .006, but not the nonseeing condition, *t*(36) = 0.56, *p* = .58. For detection criterion, Bayesian analyses presented weaker support for either main effects or interactions (centre panel of Fig. 3b; congruency, *P*_*θ*_ = .72; blindfold, *P*_*θ*_ = .66; Congruency × Blindfold interaction, *P*_*θ*_ = .93). Finally, examining response times, we observed significant speeding by congruent perspectives in *both* seeing and nonseeing conditions (thus tracking the low-level directional features of the avatar), *F*(1, 36) = 9.64, *p* = .004, in the absence of a main effect of blindfold, *F*(1, 36) = 2.63, *p* = .11, nor a Congruency × Blindfold interaction, *F*(1, 36) = 2.74, *p* = .60. Thus, response times, unlike perceptual sensitivity, were modulated by spatial congruency but were insensitive to shared perception.

In summary, the results of Experiment 3 provide evidence that the effects of the avatar on visual sensitivity are gated by beliefs about shared perception, and are not explained by low-level directional features which remained constant in the seeing and nonseeing conditions.

## “Other” Condition

The previous analyses focused on the critical “self” condition, in which the avatar was irrelevant for the perceptual response to the Gabor. In classic dot perspective tasks, the “other” condition is also of interest and used to measure whether “egocentric interference” affects judgements from the avatar’s perspective. This effect manifests as lower accuracy and slower response times in the incongruent versus congruent trials (Samson et al., 2010). We were unable to conduct a similar analysis here due to the detection task creating imbalances in this condition—in three-quarters of “other” trials (Gabor-present-incongruent, Gabor-absent-congruent, and Gabor-absent-incongruent), a “No” response is the correct answer. Only on congruent-Gabor-present trials is “Yes” the correct answer. We suspect that this feature of the “other” condition led subjects to adopt a conservative criterion such that they were more likely to say “No”, leading to faster RTs and higher accuracy for “incongruent” trials (where “No” is always the correct answer, regardless of Gabor presence).

Indeed, in Experiment 1, performance was higher overall on the “other” compared with “self” condition (main effect of perspective), *F*(1, 9) = 37.89, *p* < .001, including between Gabor-absent “other” and Gabor-absent “self” trials (see Fig. 1b), *t*(9) = 2.41, *p* = .04. The same effect on accuracy was observed in Experiment 2—an overall higher accuracy in “other” compared with “self” trials (main effect of perspective), *F*(1, 17) = 41.76, *p* < .001, as well as in Gabor-absent “other” compared with Gabor-absent “self” trials (see Fig. 2b), *t*(17) = 6.41, *p* < .001. Experiment 2 now also exhibited response time effects that were previously insignificant in Experiment 1. Response times were overall faster in the “other” than in the “self” condition (main effect of perspective, *F*(1, 17) = 26.71, *p* < .001, with Gabor-absent “other” trials having faster responses times than those in the Gabor-absent “self” trials (see Fig. 2b), *t*(17) = −5.56, *p* < .001. Additionally, “other” Gabor-present-incongruent trials also exhibited higher accuracy, *t*(17) = 6.62, *p* < .001; Perspective × Congruency interaction, *F*(1, 17) = 55.59, *p* < .001, and faster reaction times, *t*(17) = −2.30, *p* = .03; Perspective × Congruency interaction, *F*(1, 17) = 7.75, *p* = .01, compared with Gabor-present-congruent trials. Together, these findings suggest that subjects adopted a conservative criterion on “other” trials, and treated “No” as the default response.

Notably, this effect on “other” trial accuracy was only present in the seeing condition of Experiment 3 (which shows similar imbalances in response requirements as Experiment 2) and not in the nonseeing condition (see Supplementary Materials’ Fig. S2), congruent-seeing versus incongruent-seeing), *t*(36) = 7.59, *p* < .001; congruent-nonseeing versus incongruent-nonseeing, *t*(36) = 1.82, *p* =.07. In the nonseeing condition, the correct answer on “other” trials was always “No”, perhaps explaining the very high accuracy observed in this condition and the limited effects of stimulus congruency.

To directly assess whether participants adopted a conservative criterion on “other” trials, we performed an additional SDT analysis. Responses in the “other” congruent (Experiment 2) or congruent-seeing (Experiment 3) trials were sorted into hits, false alarms, misses, and correct rejections. For each subject, detection sensitivity (*d'*) and criterion (*c*) were calculated for congruent trials in Experiment 2, and for the congruent-seeing cell of the factorial design in Experiment 3. SDT analysis was not applicable to the incongruent trials of Experiment 2 or the incongruent-seeing/all nonseeing conditions of Experiment 3. This is because in these trial types, the correct answer is a “No” response despite the Gabor sometimes being present or absent, and thus standard SDT classifications of responses do not apply.

Notably, the criterion estimated in the congruent(-seeing) condition (see Fig. S4 in the Supplementary Materials) in both Experiments 2 and 3 was conservative (positive), confirming that participants were biased towards responding “No” when they were asked to judge the stimulus from the avatar’s perspective. We surmise that this prior expectation that a “No” response will be required (which is appropriate, given the unbalanced design) is likely to have led to higher correct rejection rates on the incongruent (and nonseeing) condition(s) (see Fig. S2 in the Supplementary Materials), resulting in the higher accuracy and response times observed in incongruent versus congruent trials, as well as the higher overall accuracy and response times observed in the “other” versus “self” condition.

## General discussion

Previous work has shown that the perspective of others interferes with one’s own judgments and decisions (Cole et al., 2015; Furlanetto et al., 2016; Qureshi et al., 2010; Samson et al., 2010; Wiese et al., 2012), consistent with an automatic process that represents the viewpoint and potential goals of others (Gallotti & Frith, 2013; Tuomela, 2006). Here, we ask whether social cueing also leads to shifts in visual processing. We found across three experiments that participants were more likely to (accurately) detect a near-threshold visual stimulus from their own perspective when the avatar could also see the Gabor. By introducing spatially localized null (noise-only) trials into Experiment 2, we showed this effect is driven by an increase in detection sensitivity (*d'*)—an increase in hit rate, without a change in false alarm rate. Finally, by “blindfolding” the avatar in Experiment 3, we manipulated beliefs about whether the avatar is able to see while leaving the directional features of the avatar constant. We showed that the effect of the avatar on perceptual sensitivity is only obtained when the participant believed the avatar could also see the Gabor.

Our findings cannot be ascribed to decisional or response interference because a change in performance was only observed on Gabor-present “self” trials (“hits”), and response times did not mirror the effect of congruency on sensitivity. Indeed, in Experiment 3, response times were significantly speeded by congruent perspectives in *both* seeing and nonseeing conditions (thus tracking the low-level directional features of the avatar, consistent with recent findings; Cole, Atkinson, Le, & Smith, 2016; Conway, Lee, Ojaghi, Catmur, & Bird, 2017), whereas a boost in perceptual sensitivity was only observed in the seeing condition. Taken together, our findings suggest the avatar’s viewpoint modulates early perceptual processing, thereby potentiating perception of stimuli for which others are also able to see and act upon (Otten, Seth, & Pinto, 2017; Teufel, Fletcher, & Davis, 2010).

The mechanism by which shared perception translates to enhanced perceptual sensitivity remains to be determined. An appealing hypothesis is that the avatar’s viewpoint acts as an attentional cue to prioritise particular spatial locations (Otten et al., 2017), thereby leading to top-down potentiation of perceptual sensitivity. The presence or absence of the blindfold may in turn modulate the reliability of this prior (Summerfield & Egner, 2009). However, it remains uncertain which amongst a broad class of attentional mechanisms that result in increased signal-to-noise ratio of sensory processing are altered by shared perception (Luo & Maunsell, 2015; Smith & Ratcliff, 2009). For instance, shared perception may boost signal, suppress internal noise, or both. Our findings lay the foundations for a computational and neural understanding of how social context affects perceptual processing—for instance, through application of noise-perturbation analyses (Lu & Dosher, 1998; Wyart et al., 2012) and/or pitting shared perception against manipulations of signal probability and relevance (Summerfield & Egner, 2009).

Notably, the modulation of perception by the avatar was abolished by a simple blindfold manipulation. This weakens explanations of a sensitivity change in terms of “submentalizing”, in which directional cues bias attention irrespective of the observer’s belief about whether the avatar can also see the target (Furlanetto et al., 2016; Heyes, 2014; Santiesteban, Catmur, Hopkins, Bird, & Heyes, 2014). However, we acknowledge that the blindfold may act to abolish automatic gaze following without requiring a belief about seeing to be instantiated. We think this is less likely, given that in all conditions, the participant was not able to see the avatar’s eyes, and was instead required to track (via the presence of the blindfold) whether they are in a seeing or nonseeing condition. To rule out bottom-up effects of the blindfold, future experiments could employ a belief induction manipulation in which the avatar wears either “seeing” or “nonseeing” coloured goggles (Conway et al., 2017). This ensures that the visual features of the avatars remain similar allowing changes in sensitivity to be directly attributed to the participant’s belief about whether the goggles allow seeing or not (Heyes, 2014).

We also considered the extent to which perceptual sensitivity effects observed in the “self” condition was because of interference from the “other” condition owing to “self” and “other” perspective trials being interleaved. Previous work in the perspective-taking literature has attempted to remove this possible interference by performing the dot perspective task in a wholly self-perspective condition (Samson et al., 2010). However, in noninterleaved designs, participants may perform the task without explicitly attributing a “perspective” to the avatar. Given the current controversy over the automaticity and social specificity of visual perspective-taking effects in decision-making (Cole et al., 2016; Conway et al., 2017; Furlanetto et al., 2016; Santiesteban et al., 2014), we defer this issue of the conditions that enable or disable such interference effects to future study. Instead, we make a more modest claim that when people are engaged in perspective taking (perhaps induced by interleaved trials), their perceptual sensitivity is altered on “self” trials. However, we were able to establish that the effect of the avatar on self-perceptual sensitivity was equally strong following both “self” and “other” perspective trials (see Supplementary Materials and Fig. S5)—thus, at least ruling out local task-switching or carryover effects as possible explanations of our results.

Lastly, we sought to understand the pattern of results obtained on “other” trials, in which the stimulus was judged from the avatar’s perspective. In the perspective-taking literature, participants typically find it more difficult to make judgements from the avatar’s perspective when what the avatar sees conflicts with their own perspective, supporting an egocentric interference effect (Samson et al., 2010). We were unable to make a similar inference here due to the detection task creating imbalances in this condition, such that three-quarters of “other” trials required a “No” response, and limiting the extent to which the “other” condition can be straightforwardly compared to the “self” condition. Instead, we observed that participants were overall more efficient in detecting whether a Gabor was present or absent from the avatar’s perspective. We reasoned this was due to participants adopting a conservative criterion (a bias towards saying “No”) which was advantageous for performing judgments from the avatar’s perspective.

Debates about the automaticity or social specificity of visual perspective taking typically focus on response times to clearly visible stimuli as the main dependent measure. Here, we go beyond these studies to reveal a selective effect of shared perception on perceptual sensitivity to low-contrast targets, in the absence of reliable changes in response times. Because socially dependent shifts in perception may not be readily apparent to observers via introspection, they may only become evident in controlled laboratory settings that permit psychophysical measurement. Future studies of the mechanisms supporting implicit mentalizing may wish to employ measures of perceptual sensitivity in addition to response times to provide a richer testing ground for competing theories. Furthermore, in our current experimental paradigm, we only probed visual detection in one constant location within each section of two halves of a 3-D room—future studies may attempt to investigate changes in perceptual sensitivity across the whole visual field.

Though it seems counterintuitive that social cognition should affect our perception of the world, others’ perception of a stimulus represents a powerful informational resource. In particular, a “we-mode” of processing has been suggested to filter stimuli based on whether they are in the vicinity of another individual and available for potential action, thus becoming relevant to the group’s goals (Gallotti & Frith, 2013; Tuomela, 2006). Our results shed new light on how social context leads to such changes in information processing, by shaping the sensitivity of early perceptual processes. To conclude, we reveal that shared perception enhances visual sensitivity. On this view, our perception of the world is not self-contained, but is in fact continually affected by what others can see.

## Electronic supplementary material

ESM 1 (DOCX 1404 kb)
