# Supplementary material for: Perceptual sensitivity is modulated by what others can see
Source: Atten Percept Psychophys. 2019 May 6;81(6):1979–90. doi: 10.3758/s13414-019-01724-5 (PMC6675914; doi:10.3758/s13414-019-01724-5)
Supplement: Supplementary file 1 — (DOCX 1404 kb) [file 13414_2019_1724_MOESM1_ESM.docx]

**SUPPLEMENTARY MATERIALS**

**Supporting Results**

To establish whether the effect of avatar congruency on “Self” trials was influenced by a carry-over effect from previous “Other” perspective trials, we calculated *d’* and *c* in Experiments 2 and 3 separately for trials preceded by “Self” or “Other” judgments. These parameters are shown in Figure S5. In both experiments we found main effects of current-trial congruency (all *P* < 0.05), but no interaction with previous trial (all *P* > 0.05), indicating that the effect of congruency was not an artefact of switching from one trial type to another.

We imposed stringent exclusion criteria for performance, only entering subjects into the analysis if they performed > 60% and < 90% correct on the critical “Self” trials. We found that, particularly in Experiments 1 and 2, this led to a relatively high exclusion rate despite the pre-experiment thresholding procedure adaptively adjusting the task difficulty for each subject. To establish that our results do not depend on this choice of exclusion criteria we repeated our analyses including all subjects. All key findings remained: self-other congruency increased hit rate on “Self” trials in Experiment 1 (*t*(18) = 4.34, *P* < 0.001) and Experiment 2 (*t*(28) = 5.00, *P* < 0.001), and in Experiment 3, the posterior estimate of the blindfold $\times$ congruency interaction was significantly positive ($P_{\theta>0}$ = 0.98). Figures S6-S9 reproduce the figures contained in the manuscript with the inclusion of all subjects.


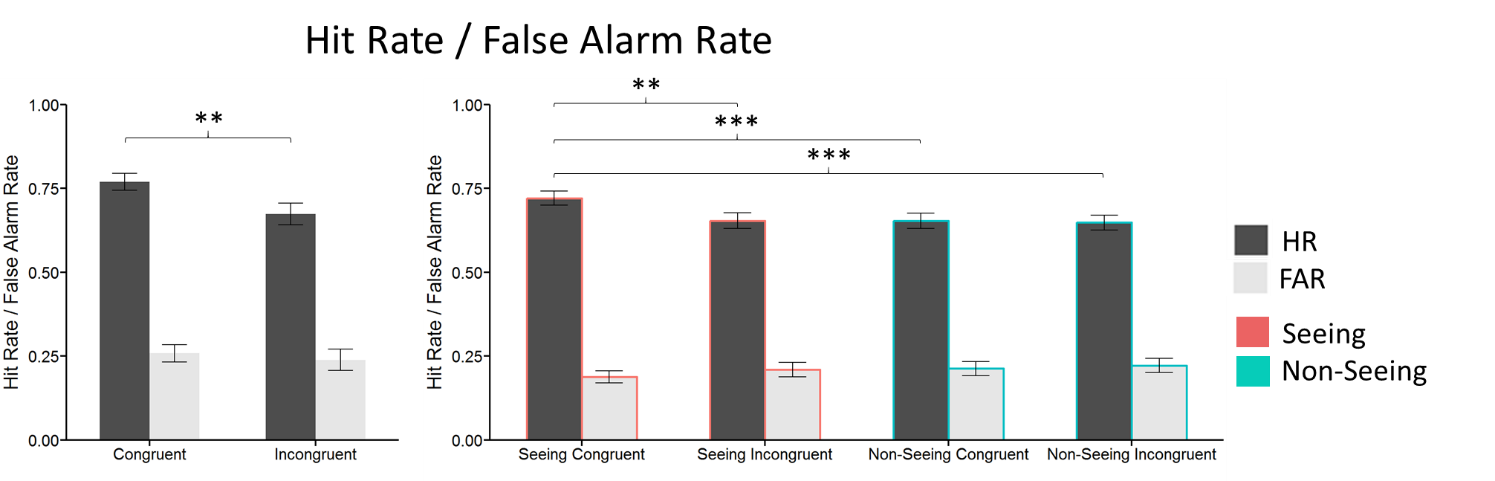


**Figure S1.** Mean hit and false alarm rates for “Self” trials in A) Experiment 2 (*N* = 18) and B) Experiment 3 (*N* = 37). Symbols indicate significance level for t-tests (* *P* <0.05, ** *P* < 0.01, *** *P* <0.001). Error bars reflect SEM.


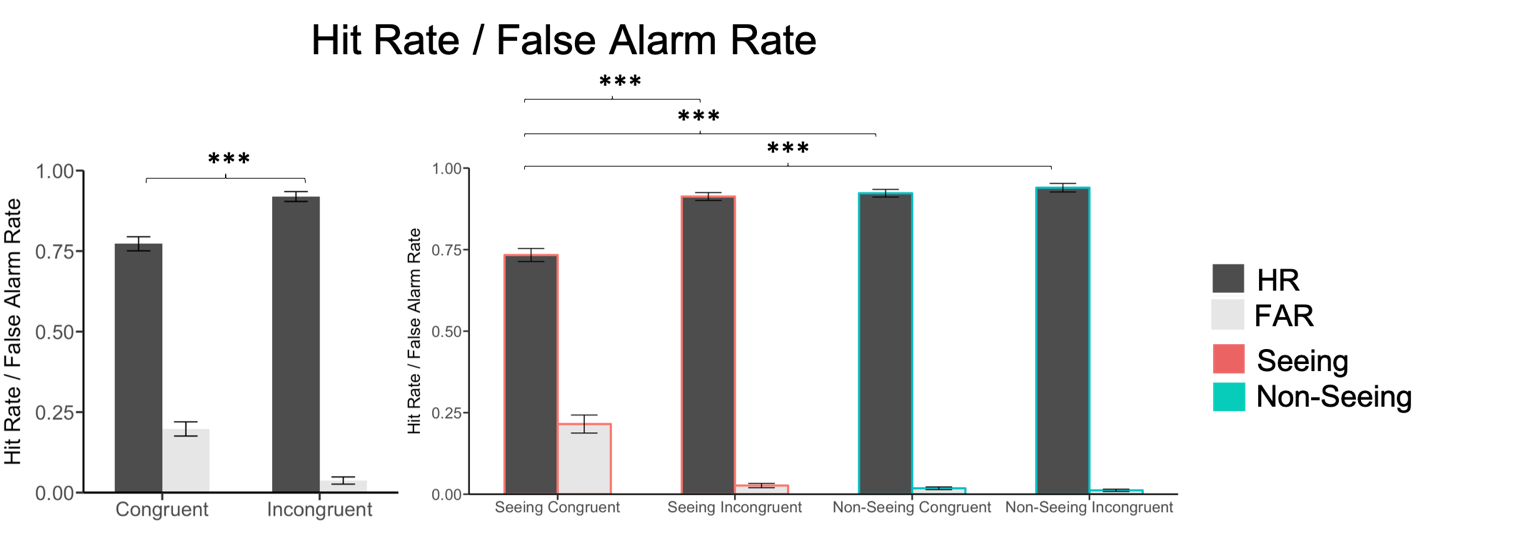
**Figure S2.** Mean hit and false alarm rates for “Other” trials in A) Experiment 2 (*N* = 18) and B) Experiment 3 (*N* = 37). On incongruent or non-seeing trials, the correct response is always to say “No”, regardless of the stimulus. This precludes standard SDT analysis. For these trial types we label saying “No” as a hit, and saying “Yes” (regardless of stimulus presence) as a false alarm. Symbols indicate significance level for t-tests (* *P* <0.05, ** *P* < 0.01, *** *P* <0.001). Error bars reflect SEM.

**
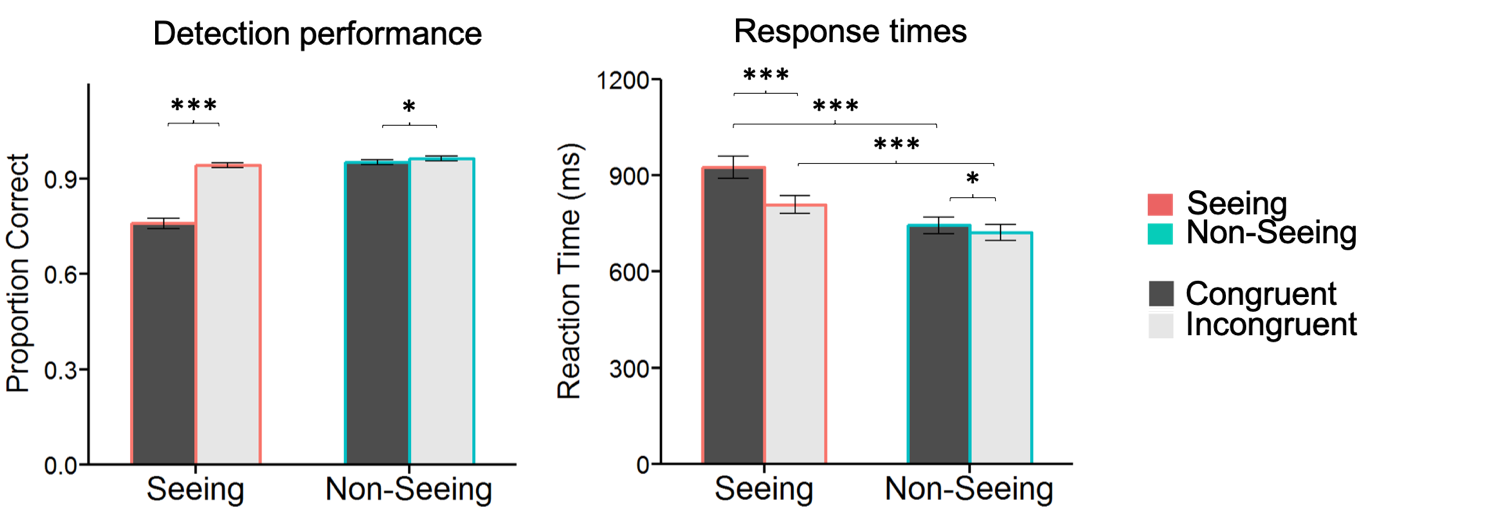
Figure S3.** Reaction times & accuracies on “Other” trials in Experiment 3 (*N* = 37). Symbols indicate the significance of paired t-tests (* *P* <0.05, ** *P* < 0.01, *** *P* <0.001). Error bars reflect SEM.


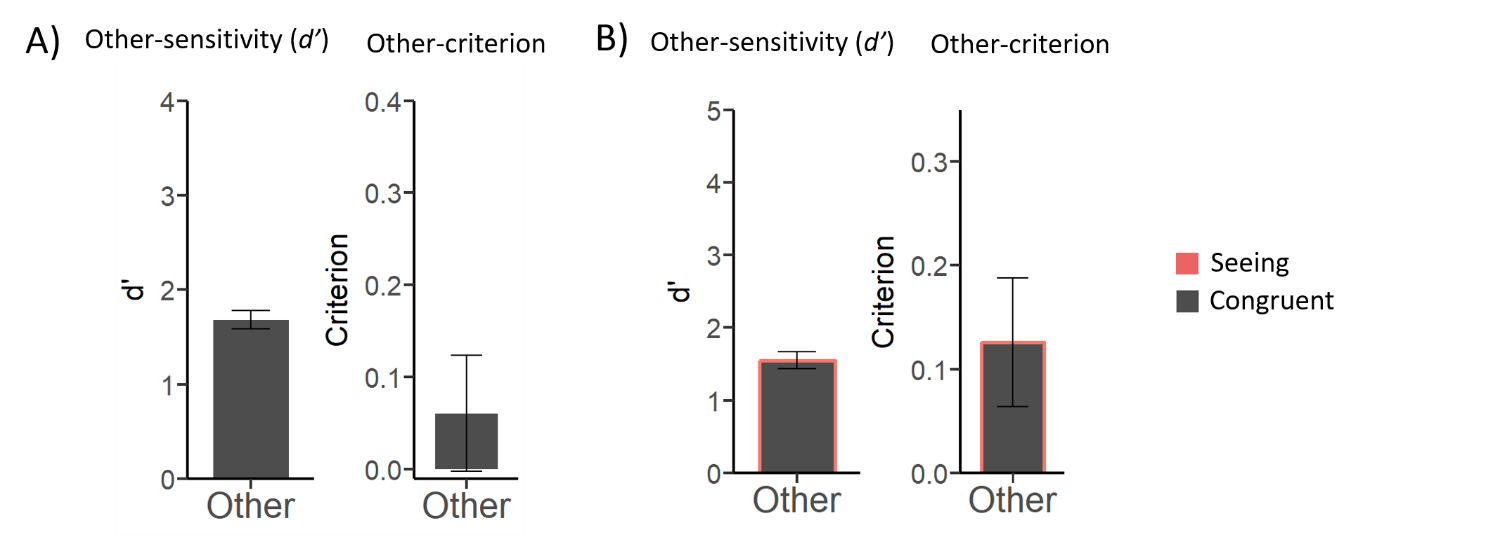


**Figure S4.** Mean *d’* and criterion for “Other” congruent trials in A) Experiment 2 and B) Experiment 3. SDT analyses were not applicable for “Other” incongruent and non-seeing-incongruent trials (see Experiment 3 Methods). Error bars indicate SEM.

**Figure S5.** Effects of previous trial identity on effect of congruency on signal detection parameters *d’* and criterion in A) Experiment 2 and B) Experiment 3.

**Figure S6.** As for main Figure 1B, without subject exclusions (*N* = 19).

**
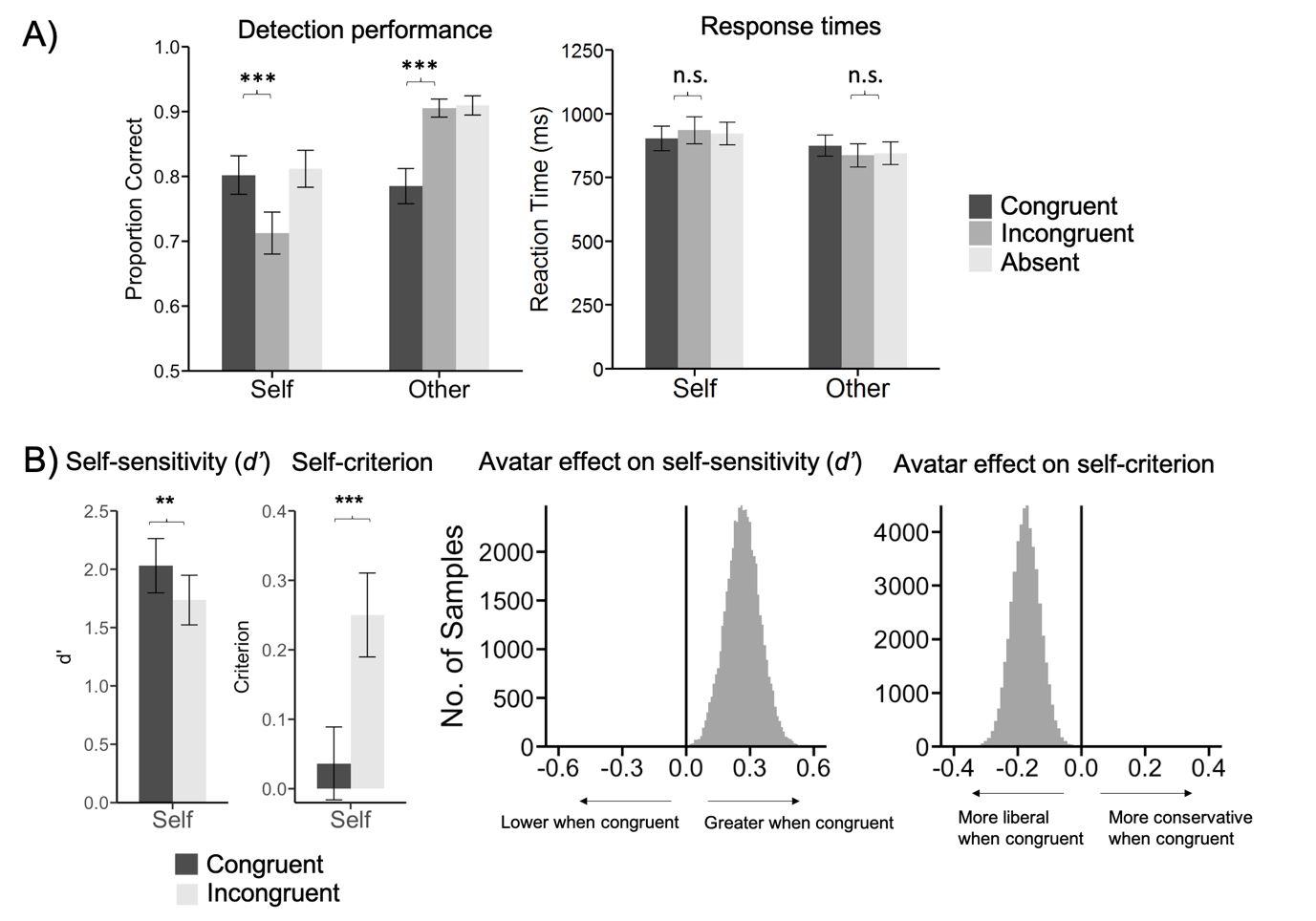
Figure S7.** As for main Figure 2, without subject exclusions (*N* = 29). For B, congruency effect on both d’ and criterion are $P_{\theta}$ = 1.00.

**Figure S8.** As for main Figure 3, without subject exclusions (*N* = 46). Bayesian analyses confirmed effects on detection sensitivity, *d’* (B; congruency, *P_θ_* = 0.96; blindfold, *P_θ_* = 0.84; congruency × blindfold interaction, *P_θ_* = 0.99). For detection criterion, weaker support was found for each main effect (congruency, *P_θ_* = 0.30; blindfold, *P_θ_* = 0.13) but revealed a congruency × blindfold interaction (*P_θ_* = 0.99) due to a significantly more liberal criterion in seeing-congruent compared to seeing-incongruent trials.


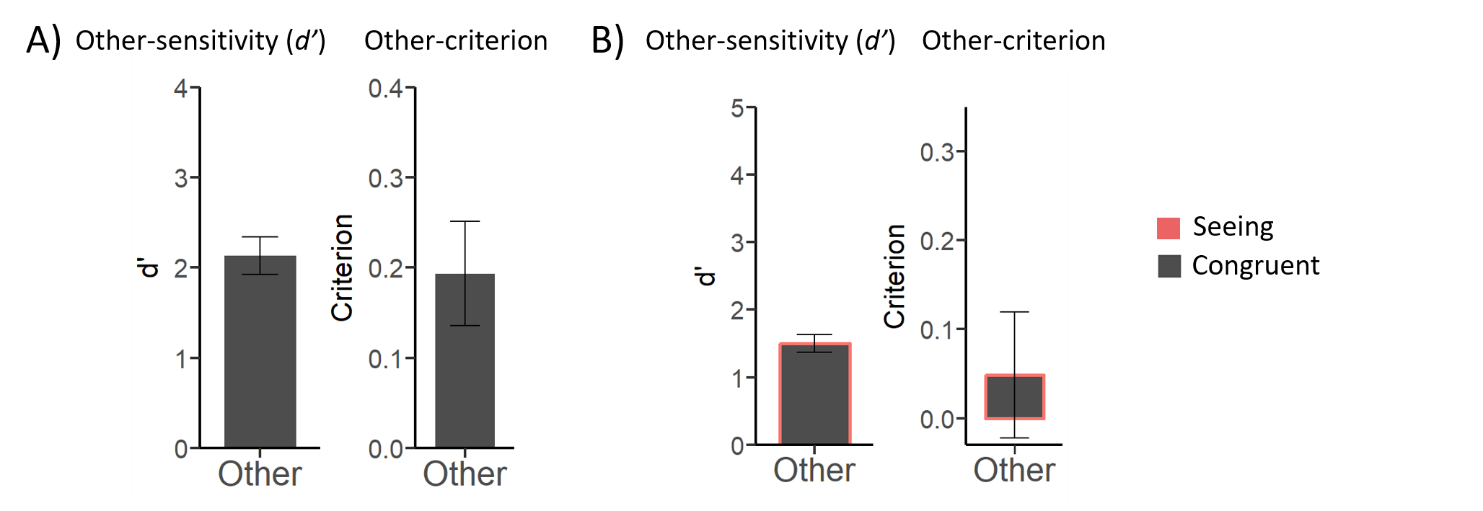


**Figure S9.** As for Figure S4, without subject exclusions: A) Experiment 2 (*N* = 29) and B) 3 (*N* = 46). Error bars indicate SEM.
